# Larval crowding effects during early development in the Chinese oak silkmoth *Antheraea pernyi* (Lepidoptera: Saturniidae)

**DOI:** 10.1002/ece3.9283

**Published:** 2022-09-11

**Authors:** Juliano Morimoto

**Affiliations:** ^1^ School of Biological Sciences University of Aberdeen, Zoology Building Aberdeen UK; ^2^ Programa de Pós‐graduação em Ecologia e Conservação Universidade Federal do Paraná Curitiba Brazil

**Keywords:** density‐dependent, garment, natural history

## Abstract

Chinese sericulture relies in part on the rearing of the Chinese oak silkmoth *Antheraea pernyi*, an insect with key cultural and ecological roles. While feeding primarily on oak, *Antheraea* species are known to accept alternative hosts such as birch *Betula sp* with little to no apparent negative fitness consequences. This opens up the range of hostplants that could be used for large‐scale rearing of *A. pernyi* for silk production and food, or used by this species in possible invasions. To date, however, the natural history and ecology of *A. pernyi* remain subject of investigation. For instance, we still do not know how individuals respond to crowding developmental environments, which is an important factor to consider for the ecology of the species as well as for commercial rearing. Here, I describe the implications of larval crowding to the survival and growth of *A. pernyi* larvae during early development. I show that higher crowding is associated with stronger negative effects on growth and survival, corroborating findings from other holometabolous insects. I then discuss the implications of this findings for our understanding of optimum larval crowding. Overall, the findings reveal important ecological information for an insect species key for provisioning and cultural ecosystem services.

## Graphical abstract

Getting data for the number of larvae in a substrate is an inglorious and surprisingly difficult task. Yet, this information is key for good ecological research and also, for maximising yield in insect growth farms. Here, I described semi‐natural responses of a silkworm to varying crowding levels, and propose a framework which can help the field infer natural range of larval crowding from laboratory experiments.

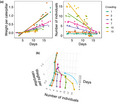

## 1 INTRODUCTION

Sericulture has been a key economic and cultural activity worldwide, and has its origin in ancient China (Altman & Farrell, 2022; Cherry, 1987). Evidence suggests that sericulture started in China more than 5000 years ago, first with the domestication of *Bombyx mori* Linnaeus (1758) (Lepidoptera: Bombycidae) and later, with the domestication of the Chinese oak silkmoth *Antheraea pernyi* Guérin‐Méneville (1855) (Lepidoptera: Saturniidae) (Cherry, 1987; Goldsmith et al., 2004). The latter has progressively become an important, albeit at times, overlooked species for silk production but also as food source and part of the traditional Chinese medicine (Li et al., 2017; Liu et al., 2010). In fact, *A. pernyi* has highly nutritional profiles and has been an important edible insect in Chinese culture (Li et al., 2020). Nonetheless, despite its economic and cultural services, many aspects of the ecology and natural history of *A. pernyi*, which can inform artificial rearing, remain to be uncovered.

Ancient texts suggest that *A. pernyi* was originally endemic of China (Li et al., 2017; Yu, 1987) and was later introduced in India, Japan, Spain, and Ukraine (Peigler, 2012). The majority of studies in *A. pernyi* have focused on its physiology. For example, in the early 1970s and 1980s, studies focused on using *A. pernyi* as model to understand the hormonal regulation of metamorphosis as well as in isolating and characterizing the antibacterial activity of Cecropins (Qu et al., 1982; Qu et al., 1987; Truman, 1973). More recently, studies focused on physiological processes involved in homeostasis and metamorphosis and have included, for instance, the identification of genes involved in sex pheromone biosynthesis (Li et al., 2005; Wang et al., 2018, 2020) and the activation of heat‐shock proteins on immune response (Liu et al., 2018). Surprisingly though, the majority of the studies in *A. pernyi* have focused on the molecular mechanisms [e.g., (Geng et al., 2017; Sun et al., 2016; Xin et al., 2017; Zhang et al., 2015)], with only limited information of its behavior and ecology above and beyond sericulture (Lounibos, 1975). This lack of a holistic ecological view of the species can hamper efforts to understand the species' response to changing climate.

**FIGURE 1:**
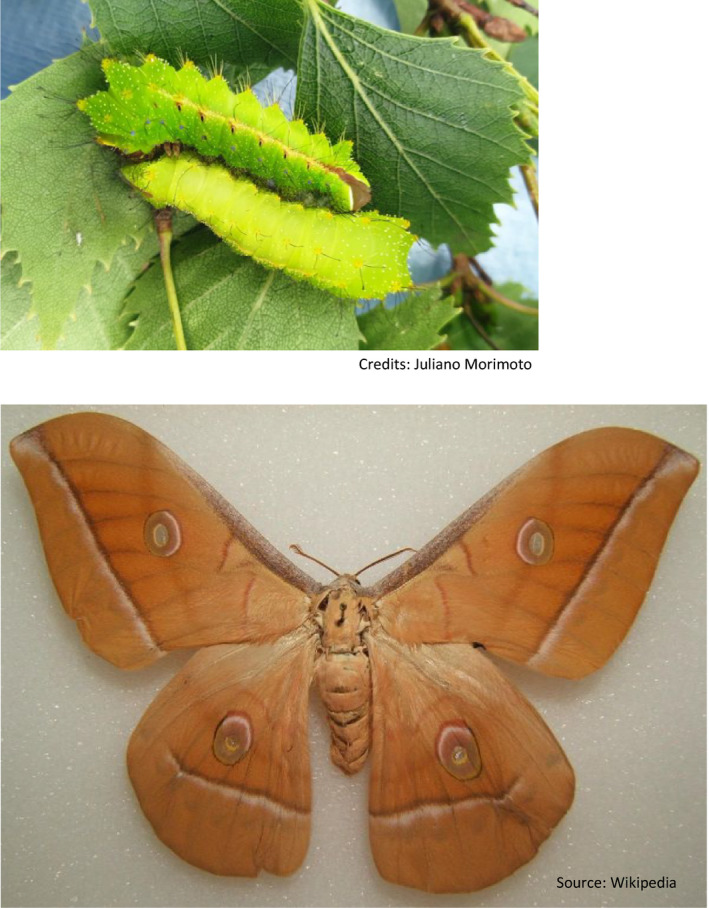
Early fifth‐instar *A. pernyi* caterpillars in the present experiment (top) and adult *A. pernyi* (source: Wikipedia) (bottom)

*Antheraea pernyi* displays an interesting gradient of voltinism (i.e., number of generations per year), and can be uni‐ or bi‐voltine depending on the latitude, with southern populations adopting the former strategy while most northern, the latter (Qin et al., 2003). A transition region, where voltinism is dynamic, occurs at ca. 35°–36° N (Li et al., 2017). Each female lays a bout of 200–400 eggs, each with ~2.2 mm diameter and coated with a colloid substance, which gives its brown coloration (Li et al., 2017). As with other insects, there is no description of larval crowding levels of *A. pernyi* in nature. Nevertheless, it is known that larval development has six instars, whereby the first consists of small larvae that are black in coloration followed by a progressive transformation towards a large vibrant lime‐green, yellow, or red‐orange caterpillars (see, e.g., Figure 1), depending on the variety of *A. pernyi* (Li et al., 2017; Qin et al., 2003). Reports suggest that larval development can last for up to 55 days at an average temperature of 24°C (Qin et al., 2003). Silk production occurs as a result of pupation (Lounibos, 1975), which can last up to 5 days. Interestingly, *A. pernyi* larvae can undergo a long diapause period after cold exposure or develop into adults without diapause, a decision that is based on the monitoring of day lengths by the larvae (Williams & Adkisson, 1964). *Antheraea pernyi*, as other species of the genus, resort to the secretion of an enzyme known as cocoonase along a protease solution that digest the sericin and enable the adult to emerge (Truman, 1971). It is important to mention that *A. pernyi* pupae and the developing adult display a characteristic pattern of abdominal movement up until adult emergence, with simple movements at 1–1.5 h intervals at first up until a burst of abdominal movement characteristic of Saturniid moths soon prior to adult eclosion (Truman, 1971). Adult *A. pernyi* are large, with female wingspan reaching up to 16 cm. Sexual dimorphism in body size has been reported whereby females are larger than males (Li et al., 2017; Qin et al., 2003), a pattern that is consistent with other insects (e.g., Esperk et al., 2007; Teder & Tammaru, 2005) (Figure 1). While feeding primarily on oak, *A. pernyi* is known to accept birch as an alternative food source with little or no apparent negative fitness consequences (Li et al., 2017; Qin et al., 2003) [cf. (Stekolnikov, 2012)].

In this study, I measured the effects of larval crowding on early‐life growth rate and survival in *A. pernyi*. This study contributes toward our understanding of the basic ecology of the species, which might lead to useful information for sericulture. For instance, as in other economic activities, silk yield is dependent upon the number of larvae that can be grown per unit of area of suitable land, where a trade‐off needs to be achieved between the crowding of the larvae per unit of space and the expected growth rate of the individuals, particularly early in life, in order to maximize turnaround time for silk production during pupation. Yet, as with other insects (see, e.g. Morimoto & Pietras, 2020), virtually nothing is known on the effects of larval crowding on growth rates early in life of this species. I housed groups of *A. pernyi* of varying sizes in birch, an alternative yet ubiquitous food source for *A. pernyi*. This choice of hostplant was based on the information of the stock population that the eggs were sourced, which were maintained in birch. I measured growth rate and survival for the first 17 days of the caterpillars' life, in order to estimate the effects of crowding during early development. Based on the literature of other insects (see review by Than et al., 2020, see also below), I predicted that larval survival and growth rate would decrease as crowding levels increased, highlighting a negative density‐dependent response to crowding. The findings of this study provide information about the effects of crowding in this economic and culturally important species, helping reveal the ecological aspects of the species' biology that can aid sericulture.

## 2 MATERIAL AND METHODS

### 2.1 Experimental protocol

In August 2021, a total of 300 *A. pernyi* eggs were sourced from a commercial supplier (Stuart Butterflies®, UK), from which parent generations were maintained in birch as hostplant. Eggs were placed in at 28°C in a plastic box (32.9 × 24.6 × 12.5 cm; 750 ml) with a soaked piece of paper and 20 g (dry weight) of freshly collected birch leaves and allowed to feed until all eggs hatched. I randomly selected first instar individuals for the following crowding treatments: 1, 2, 5, 7, 10, and 15 individuals per plastic box each with four replicates. All replicates were maintained at 28°C with a soaked piece of paper and freshly collected birch leaves for the entire 17 days of duration of the experiment. Birch branches were manually collected from young trees in Seaton Park, Aberdeen (57.1724°N, 2.0997°W) every 3 days. On average, 4.98 ± 1.035 grams of fresh birch leaves were provided at days 0, 3, 7, 11, 14, and 17, and caterpillars were allowed to develop freely (Table 1). The short interval between fresh leaves was added to the replicates ensured that caterpillars were not restricted in the availability of food, and reminiscent leaves were observed in the exchange days confirming that food was not limited. There was no difference on the amount of food provided across treatments and throughout the experiment (Crowding: *F*_6,88_ = 0.413, *p* = .868; Day: *F*_1,88_ = 0.047, *p* = .828; Crowding*Day: *F*_6,88_ = 1.415, *p* = .217). Fresh moist paper was also exchanged along with birch leaves to ensure that humidity was maintained in the boxes. I scored the number of individuals alive and the *per* individual body mass of the caterpillars in each treatment and replicate at each of the timepoints (days). Body mass was measured using a Sartorius® Secura 125‐1S balance (0.01 mg precision). For lower crowding treatments, individuals were studied from egg to early fifth‐instar (out of six) larval stage, whereas for higher densities, individuals died before fifth instar. Therefore, larval development was considered “continuous” (i.e., increasing weight) rather than discrete (i.e., number of instars). This approach was useful to avoid biases introduced by, for example, averaging the instars in a treatment due to the potential increase in variation on the timing that each individual transition to a different instar (particularly in high crowding treatments).

**TABLE 1:** Average food provided per crowding treatment throughout the experiment. Fresh food was provided at days 0, 3, 7, 11, 14, and 17 (see Section 2)

Crowding treatment	Mean (g)	SD
0 (control)	5.12	1.26
1	4.95	1.07
2	5.00	1.17
5	4.78	0.89
7	5.17	1.11
10	5.09	1.05
15	4.69	0.79

### 2.2 Statistical analyses

Data were analyzed in R 4.1.3 (R Core Team, 2019). To test the growth rate (i.e., weight gained over time) and the effects of larval crowding, I fitted a mixed linear regression using the lmer function of the “lmerTest” package with the single and interactive effects of days and crowding as independent variables, replicate ID nested with days to account for repetitive measurements as random variable, and either *per* individual weight or number of individuals as dependent variables. I used the “ggplot2” package for data visualization.

## 3 RESULTS

**FIGURE 2:**
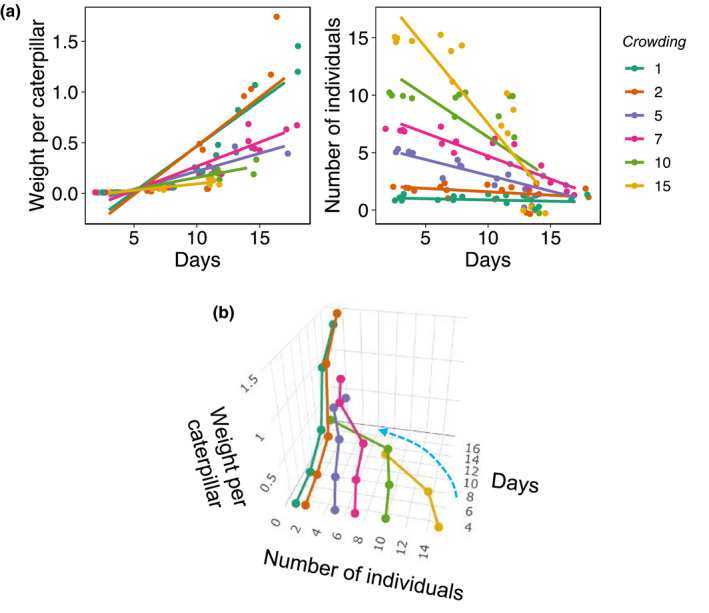
*A. pernyi* responses to larval crowding early in life. (a) Weight per caterpillar (left) and number of individuals alive per group (right) over the 17 days experiments, across groups of different crowding levels. (b) 3D plot of weight per caterpillar, number of individuals alive in the group, and time to highlight the fast corrective trajectory (blue arrow) in survival of groups that are farther from the crowding with highest growth towards survival of individuals that matched that of the fastest growing group.

There was a statistically significant interaction between crowding and days on both *per* individual weight (*F*_1,66.156_ = 14.370, *p* < .001) and number of individuals (*F*_1,79.640_ = 43.661, *p* < .001) meaning that the negative effects of crowding were more accentuated in more crowded (possibly *over*crowded) environments. In particular, caterpillar growth over time was progressively more decelerated as crowding levels increased (Figure 2a). Likewise, the number of caterpillars alive declined sharply in more versus less crowded groups (Figure 2a). A three‐dimensional plot shows the speed at which the number of individuals decline toward the lowest densities (e.g., blue arrow in Figure 2b), not only corroborating the strongest effects of highest densities but also suggesting that, under the conditions of the experiment, the lowest densities were more likely to reflect the best developmental environments. This is interesting because, as discussed below, it can be an important way in which optimum natural history densities can be inferred from natural population under experimental conditions (see Section 4 for more detailed discussion).

## 4 DISCUSSION

Larval crowding is a known factor shaping life histories across insect species (Than et al., 2020) and playing an important role in the design of artificial rearing systems (Leppla & Ashley, 1989). Here, I investigated the effect of larval crowding on growth and survival of early stages of development in the Chinese oak silkmoth *A. pernyi*, a species with ancient economic and cultural significance in sericulture, food source, and traditional Chinese medicine. Under the conditions of the experiment, I showed the more pronounced negative effects of crowding on larval growth and survival over time as larval crowding increased from one to 15 larvae per group. The findings contribute to our understanding of the ecology of *A. pernyi* by showing how caterpillars respond to varying crowding levels during development early in life, an aspect that has been relatively neglected over the last decades of studies focusing primarily on the species' physiology. Importantly, as an economically important species, the findings here can stimulate future studies on the fitness effects of larval crowding throughout development, formalizing a framework that can be implemented by farmers to maximize silk yield and minimize the impacts of crowding cultures.

This report focused on early life stages, that is, the first instars of larval development and the findings agree with broader literature. For instance, I found that growth rate was significantly reduced, with magnitude of the effect increasing (i.e., being more negative) as larval crowding increased. Similar results of the lasting effects of early life crowding were found in the *Epirrita autumnata* Borkhausen (1794) (Lepidoptera: Geometridae) whereby early life crowding resulted in shorter growth periods and achieved overall lower weight in the last instar of development (Tammaru et al., 2000). Likewise, a negative relationship between larval survival and weight with larval crowding was also found in *Earias vittella* Fabricius (1794) (Lepidoptera: Noctuidae) (Tripathi & Singh, 1990) and cabbage moth *Mamestra brassicae* Linnaeus (1758) (Lepidoptera: Noctuidae) (see also Than et al., 2020 and references therein). Thus, the results presented here confirm previous findings in the literature on density‐dependent effects on larval development in insects, and suggest that larval crowding during early stages of development decrease growth and survival, with likely implications to the life histories of the adult as well as, from an economic perspective, lower silk yield. It is important to note that, particularly in Lepidopterans, density‐dependent effects can improve survival via enhanced immunity (“density‐dependent prophylaxis”) (Cotter et al., 2004). An example is the increased immune and antioxidant activity upon larval crowding of the northern armyworm *Mythimna separata* Walker (1865) (Lepidoptera: Noctuidae) (Li et al., 2021). Similar increases have been shown under specific circumstances in other insect groups such as flies as a physiological process known as hormesis (Henry et al., 2018; Lushchak et al., 2019). Future studies building upon the knowledge of the immune system of *A. pernyi* in response to crowding will shed light into potential density‐dependent prophylaxis in this species.

**FIGURE 3:**
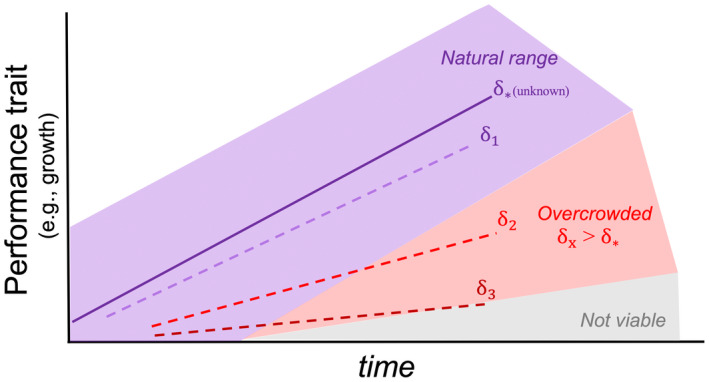
Framework to indirectly assess natural ranges of crowding in laboratory experiments. Assuming that responses to crowding evolve by selection, then there are crowding levels, which are optimum for individual development. Directly measuring these natural ranges of crowding levels may be cumbersome or, in some cases, impossible. However, the magnitude of the (negative) effect of larval crowding on traits may enable us to quantify the mean and range of crowding levels that are optimum. This framework infers the natural range of crowding levels based on the different of slope of the performance trait (e.g., growth) over time between crowding levels. Crowding level $\delta_{x}$ > $\delta_{*}$ (overcrowding) result in negative effects proportional to the distance between $\delta_{x}$ and $\delta_{*}$. As $\delta_{x}$ approaches $\delta_{*}$, the slopes would converge. Nonviable regions are regions where crowding is too high and unviable. It is also possible that, for lower densities than the optimum ($\delta_{x}$ < $\delta_{*}$, or undercrowding), slope could be either unaffected by density or negatively affected by density (i.e., Allee effect) (not displayed in the figure for clarity).

I found that the magnitude of the negative effect of larval crowding on growth and survival increased as larval crowding increased (e.g., Figure 2). In particular, a three‐dimensional plot shows that larval survival (as the number of individuals alive in the treatment) seemed to form a trajectory toward lower crowding levels (e.g., blue arrow in Figure 2b), which was more accentuated for larval crowding treatments that deviated more strongly from lower crowding. Assuming this “gravitational pull” toward lower crowding levels reflects the natural history of the species, the analysis shows a potential way in which natural larval crowding could be inferred from experimental studies. If larval developmental responses to crowding evolve by selection, there are crowding levels $\delta_{*}$ that are optimum for individual development. Any crowding level $\delta_{x}$ above $\delta_{*}$ result in negative effects on development which are proportional to the distance between $\delta_{x}$ and $\delta_{*}$ (i.e., density‐dependent effects are stronger the more crowding levels increase or decrease). Likewise, the negative effects of crowding progressively decrease as $\delta_{x}\;$approaches $\delta_{*}$ (i.e., density‐dependent effects disappear as crowding approaches optimum). If we take two (substantially different) crowding levels $\delta_{1}$ and $\delta_{2}$ and estimate individual performance (e.g., growth) over time, we can then compare the difference between the slopes of the performance trait and time for each crowding level $\delta_{1}$ and $\delta_{2}$. The magnitude of this different in slope can reflect how far $\delta_{1}$ and $\delta_{2}$ are from the optimum crowding level $\delta_{*}$. Figure 3 shows a schematic example of this concept assuming a linear effect of crowding (although nonlinear effects are also possible, if not likely). This is a generalized framework to the data presented here in Figure 2. Note that the slope of caterpillar weight is steeper for crowing levels 1 and 2 but substantially shallower for crowding levels 5 or above. This would suggest that, in this experimental setup, optimum growth conditions for this species lies somewhere between 1 or 2 caterpillars per group. Therefore, using this framework, it may be possible to estimate the average (and potentially, the upper and lower bounds) of naturally occurring larval crowding levels that a given population of insects have evolved. This is significant because as shown previously for *Drosophila melanogaster*, laboratory studies manipulating crowding are likely to misestimate the ecological significance of the responses, either by using ranges that do not cover the natural history of the species, or by using crowding levels that are extremely high (or low) relative to the crowding levels that a species has evolved (Morimoto & Pietras, 2020). The approach proposed here provides a methodology for studying how optimum larval crowding evolves (upon domestication or in natural populations) independent of direct observations of larval densities, which can be cumbersome or in some cases, impossible to obtain for a given species. Note that the approach is likely to be valuable for wild populations, as domestication is known to alter life histories in insects (see Pérez et al., 2021 and references therein) The present experiment enclosed individuals in an artificial container that is likely too distinct from the natural environment for conclusions as to whether the data support this framework and thus, future studies will focus on formalizing and validating the framework.

## 5 CONCLUSIONS

Here, I presented a study of the responses to early life crowding on the larval growth and survival across replicate groups of *A. pernyi*, an insect species with ancient history of domestication and cultivation for silk production. The study focused on the first stages of larval development and corroborates findings from previous literature in Lepidopterans. I also proposed a simple framework derived from the results observed here to aid future experimental work investigating the evolution of life‐histories responses to larval crowding. Future studies building upon the findings shown here will have important ecological and economic implications to *A. pernyi* and more broadly, to sericulture, given that sericulture plays an important role in worldwide economy and culture.

## AUTHOR CONTRIBUTIONS

**Juliano Morimoto:** Conceptualization (equal); data curation (equal); formal analysis (equal); investigation (equal); methodology (equal); project administration (equal); resources (equal); supervision (equal); validation (equal); visualization (equal); writing – original draft (equal); writing – review and editing (equal).

## ACKNOWLEDGMENTS

The author would like to thank Dawn Shewring, Lauren Dingle, Sue Phillips and the entire team of technicians of the School of Biological Sciences for the support in the experiment. The author would like to thank Mark Paterson and Joy Molyneaux for the invaluable assistance and stimulating discussions about plants.

## FUNDING INFORMATION

JM receives support from the Royal Society Research Grant (RGS\R2\202220) and from BBSRC Responsive Mode grant (BB/V015249/1).

## CONFLICT OF INTEREST

The author has no conflict of interest to declare.

## Supporting information


Appendix S1

## Data Availability

The raw data and R code to reproduce the analysis are given as supplementary material.
